# A Pectic Polysaccharide from Sijunzi Decoction Promotes the Antioxidant Defenses of SW480 Cells

**DOI:** 10.3390/molecules22081341

**Published:** 2017-08-12

**Authors:** Chao Huang, Zhongkai Zhu, Xiyue Cao, Xingfu Chen, Yuping Fu, Zhengli Chen, Lixia Li, Xu Song, Renyong Jia, Zhongqiong Yin, Gang Ye, Bin Feng, Yuanfeng Zou

**Affiliations:** 1 Laboratory of Experimental Animal Disease Model, College of Veterinary Medicine, Sichuan Agricultural University, Chengdu 611130, China; huangchao@sicau.edu.cn (C.H.); caoxys@hotmail.com (X.C.); chzhli75@163.com (Z.C.); 2 Natural Medicine Research Center, College of Veterinary Medicine, Sichuan Agricultural University, Chengdu 611130, China; zhongkaizhu6@163.com (Z.Z.); yupingfu424@163.com (Y.F.); lilixia905@163.com (L.L.); songx@sicau.edu.cn (X.S.); yinzhongq@163.com (Z.Y.); gangyee206@163.com (G.Y.); 3 Key Laboratory of Animal Disease and Human Health of Sichuan Province, College of Veterinary Medicine, Sichuan Agricultural University, Chengdu 611130, China; jiary@sicau.edu.cn; 4 Animal Nutrition Institute, Sichuan Agricultural University, Chengdu 611130, China; fengbin@sicau.edu.cn; 5 Key Laboratory of Crop Ecophysiology and Farming System in Southwest China, Ministry of Agriculture, College of Agronomy, Sichuan Agricultural University, Chengdu 611130, China; chenxf64@sohu.com

**Keywords:** Sijunzi Decoction, SJZD, pectic polysaccharide, oxidative stress, antioxidant defense

## Abstract

Sijunzi Decoction (SJZD) is a formula used for the treatment of spleen deficiency and gastrointestinal diseases in Traditional Chinese Medicine. Polysaccharides are reported to be the main components of SJZD responsible for its bio-functions. However, highly purified and clearly characterized polysaccharides from SJZD are not well described. Here we obtained a purified polysaccharide (SJZDP-II-I) from SJZD using ion exchange chromatography and gel filtration. Structure analysis by FT-IR and NMR identified SJZDP-II-I as a typical pectic polysaccharide with homogalacturonan and rhamnogalacturonan type I regions and arabinogalactan type I and II as side chains. In vitro studies indicated that SJZDP-II-I treatment could significantly enhance the total antioxidant capacity of SW480 cells, resulting from the promoted expressions of antioxidant enzymes and their master regulator PGC-1α, which would be valuable for further research and applications.

## 1. Introduction

As a conventional Traditional Chinese Medicine Formula (TCMF), Sijunzi Decoction (SJZD), consisting of four crude herbs (*Panax ginseng*, *Atractylodes macrocephala*, *Poria cocos* and *Glycyrrhiza uralensis*) in the ratio of 3:3:3:2, has been widely applied for over one thousand years in the treatment of gastrointestinal diseases, such as stomach aches, rugitus, nausea, vomiting and diarrhea [1,2]. Modern pharmacological studies have further revealed its potential effects on the immune system activation and cancer treatment [1,3,4]. Due to the expensive price of *Panax ginseng*, *Codonopsis pilosula*, with similar biological activities, has been used as a substitute for more than hundreds of years in SJZD [5,6]. Flavonoids, coumarins, triterpenoid and polysaccharides were reported to be the main components of SJZD [7], and polysaccharides were considered as the most abundant and effective component [4].

The functions of polysaccharides from each herb of SJZD were widely reported. *C. pilosula* polysaccharides showed several bioactivities, such as enhancement of lymphocyte proliferation [8], improvement of the compensatory hematopoiesis of spleen [9], antitumor activity [10], and immunomodulating activity [11]. Additionally, polysaccharides from *A. macrocephala* were reported to regulate the gut microbes and were widely used in the treatment of chronic intestinal diseases [12]. *P. cocos* and *G. uralensis* polysaccharides showed antioxidant and anti-infectious capacity, respectively [13,14]. However, highly purified polysaccharides from SJZD with clearly characterized structures are not well described. Han et al. have found that the total polysaccharides from SJZD could enhance the proliferation of intestinal epithelial cells [15]. Liu et al. have also reported that the total SJZD polysaccharides could improve intestinal restitution and protect against indomethacin-induced damage of intestinal epithelial cells [16]. Three major SJZD polysaccharide fractions were isolated by Wang et al., the monosaccharide components of which were characterized, but further structural information remains unclear [17]. Therefore, it is of interest and necessity to deepen the study of the structural properties of polysaccharides from SJZD.

Reactive oxygen species (ROS), widely generated during life processes, are involved in gene transcription signal transduction, and regulation of the soluble guanylate cyclase activity of cells [18]. The concentration of ROS is precisely controlled by antioxidants, both enzymatic and non-enzymatic. When the balance between ROS production and antioxidant defenses is broken, excessive ROS accumulation may result in progressive oxidative damage to lipids, proteins and DNA, which is collectively referred to as ‘oxidative stress’ [19]. The intestine is vulnerable to oxidative stress, due to its constant exposure to diet-derived oxidants, mutagens, and iron salts as well as endogenously generated ROS [20]. Therefore, antioxidant defenses are critical for intestinal functions, impairment of which is widely involved in intestinal disorders, such as inflammatory bowel diseases and intestinal absorption dysfunctions [21,22], making the identification of molecules that can promote the antioxidant capability in intestine very important.

In this work, ion exchange chromatography and gel filtration were used to purify polysaccharides from SJZD, and three fractions—SJZDP-I-I, SJZDP-I-II, SJZDP-II-I—were obtained. Characterization was performed based on bioassay guidance, from which SJZDP-II-I was selected. Structural analysis by FT-IR and NMR identified SJZDP-II-I as a typical pectic polysaccharide with HG regions, RG-I regions and AG-I/AG-II as side chains. Further, we have found that the treatment with SJZDP-II-I could promote the expressions of antioxidant enzymes and their master regulator PGC-1α, resulting in significant enhancement of the total antioxidant capacity of SW480 cells. These findings reveal a new perspective on the role of the pectic polysaccharides from SJZD in the oxidative defenses, which should elicit interest for further applications.

## 2. Results and Discussion

### 2.1. Fractionation of Pectic Polysaccharides from SJZDP

**Figure 1:**
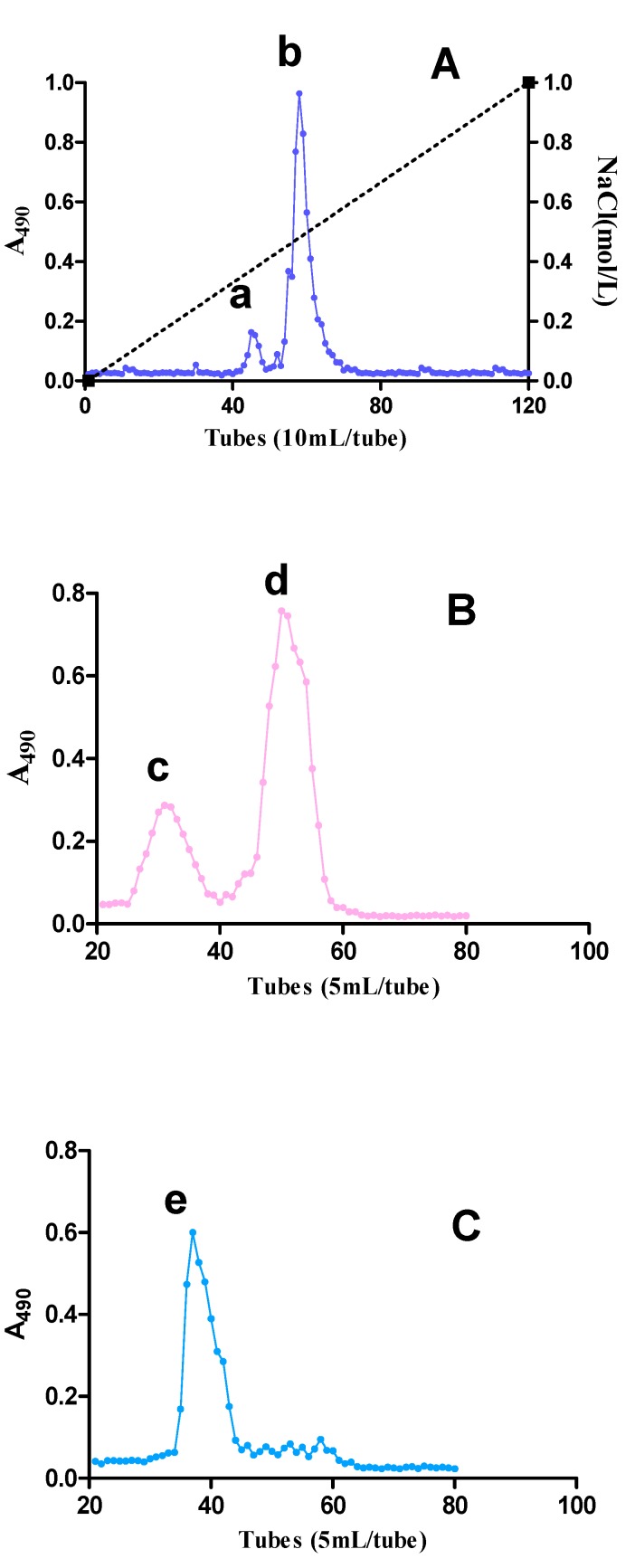
The carbohydrate elution profiles were monitored using the phenol–sulfuric acid assay (A490 is the absorbance at 490 nm). (**A**) Ion exchange chromatography elution profile of SJZDP: (a) SJZDP-I and (b) SJZDP-II; (**B**) Gel filtration elution profile of SJZDP-I: (c) SJZDP-I-I and (d) SJZDP-I-II; (**C**) Gel filtration elution profile of SJZDP-II: (e) SJZDP-II-I.

The crude water extracted SJZDP (200 mg) was subjected to anion exchange chromatography, whereby two acidic fractions, SJZDP-I (25 mg) and SJZDP-II (54 mg), were obtained. These two acidic fractions were further fractionated using gel filtration. After separation on a gel filtration column, SJZDP-I-I (8 mg) and SJZDP-I-II (13 mg) were obtained from SJZDP-I, and one fraction (SJZDP-II-I, 41 mg) was purified from SJZDP-II (Figure 1). SJZDP-II-I was chosen for structure elucidation as the other two fractions showed no activity in promoting the antioxidant defenses of SW480 cells (data not shown).

### 2.2. Chemical Composition of Polysaccharide

The monosaccharide composition of the isolated polysaccharide fractions was determined by GC analysis after methanolysis and TMS-derivatization. As shown in Table 1, the monosaccharide compositions of the three isolated fractions were quite different.

Fraction SJZDP-I-I was mainly composed of arabinose (Ara), galactose (Gal) and glucose (Glc), accounting for 86.0 mol % of the total carbohydrates, while the fraction SJZDP-I-II mainly consisted of Ara (18.9 mol %) and Glc (66.9 mol %). Compared to fraction SJZDP-I-I and SJZDP-I-II, the fraction SJZDP-II-I contained a higher amount of uronic acids. The fraction SJZDP-II-I was mainly composed of Ara, Gal and GalA, which accounted for 90.7 mol % of the total carbohydrates. The differences in monosaccharide composition between fractions SJZDP-I-I, SJZDP-I-II and SJZDP-II-I may explain the different activities in promoting the antioxidant defenses of SW480 cells. The obtained monosaccharide compositions of the polysaccharide fractions are different from those of previous reports, as SJZPS-Vb-1 and SJZPS-Vb-2 were mainly composed of Glc, Gal and mannose (Man) [23], and such differences may result from the different methods of extraction and purification. As SJZDP is a mixture of four herbs, the obtained monosaccharide compositions of polysaccharides may derive from different herbs. The monosaccharide Ara, Gal, Rha, Man and xylose (Xyl), may come from any of the four herbs, since the polysaccharides from all four of these herbs contained these monosaccharides [11,24,25,26]. Additionally, a previous report on polysaccharides from *P. cocos* indicated that the Glc present in SJZDP came mainly from *P. cocos* [27]. 

Size exclusion chromatography using dextran standards was applied to determine the average molecular weight of the polysaccharide fractions obtained. The results indicated that the fraction SJZDP-I-I (351.0 kDa) has the highest Mw, followed by SJZDP-II-I (171.9 kDa) and SJZDP-I-II (16.9 kDa). The Mw of these three fractions were different from those of polysaccharides SJZPS-Vb-1 (38.3 kDa) and SJZPS-Vb-2 (28.0 kDa) as previously reported [23]. 

### 2.3. Determination of the Glycosidic Linkages

The purified fraction SJZDP-II-I was analyzed for the types of glycosidic linkages using GC-MS after conversion to partially methylated alditol acetates (Table 2). 

As shown in the table, fraction SJZDP-II-I contained linkage units typical of pectic polysaccharides. The presence of 1,4- linked GalA indicates the presence of a homogalacturonan (HG) backbone, while 1,4-linked GalA with 1,2 and 1,2,4-linked Rha indicates a rhamnogalacturonan I region (RG-I) in the polymers [28]. The highly branched RG-I is known as the “hairy region” of the pectin, which normally consists of branching chains, such as arabinans, galactans and/or arabinogalactans (AG) [29]. Fraction SJZDP-II-I contained a high amount of 1,4-linked Gal units, suggesting that the polymers are rich in arabinogalactan type I (AG-I) structures, and the presence of 1,3-; 1,6-; and 1,3,6-linked Gal indicates the presence of arabinogalactan type II (AG-II) structures. The linkage units of fraction SJZDP-II-I were different from those of fractions SJZPS-Vb-1 and SJZPS-Vb-2 from SJZD, as they contain different monosaccharide compositions [23]. However, for the linkage types of Gal, fraction SJZDP-II-I contained similar linkage types with fractions SJZPS-Vb-1 and SJZPS-Vb-2, such as terminal linked, 1,6-linked and 1,3,6-linked Gal. The linkage type of fraction SJZDP-II-I was similar to those of polysaccharides from *C. pilosula*, but with a different ratio [11].

### 2.4. Structural Features

**Figure 2:**
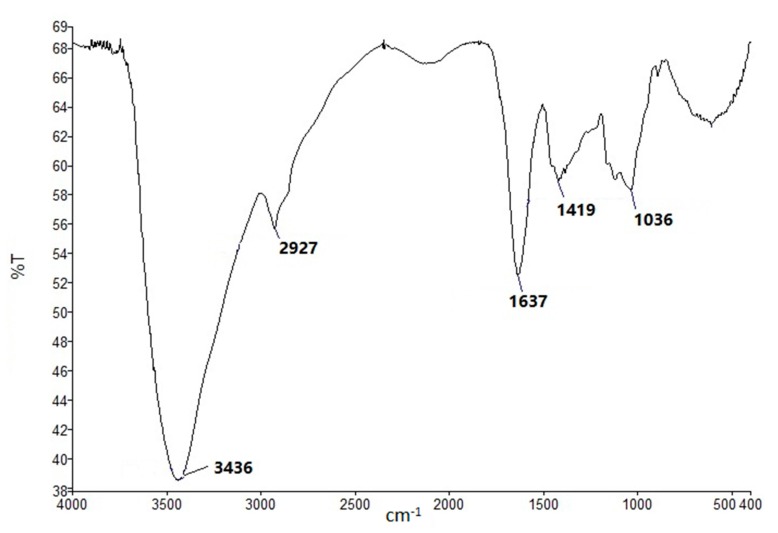
FT-IR spectra of fraction SJZDP-II-I.

As shown in Figure 2, the FT-IR chromatogram of fraction SJZDP-II-I showed characteristic absorptions of polysaccharides [30]. The absence of absorption bands at 1735 cm^−1^ and 1250 cm^−1^ indicated that the GalA units of fraction SJZDP-II-I were not esterified [31,32]. These were different from the polysaccharides isolated from *C. pilosula* [11] and *G. uralensis* [26], while polysaccharides from *C. pilosula* contain bands at 1735 cm^−1^ and 1250 cm^−1^, and polysaccharides from *G. uralensis* only contain a 1735 cm^−1^ band.

**Figure 3:**
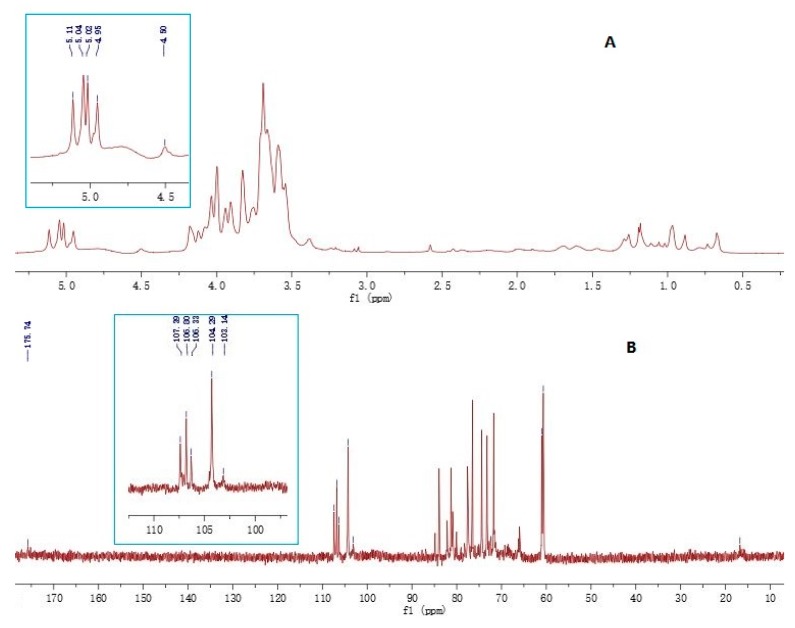
The ^1^H- and ^13^C-NMR spectra of polysaccharide fraction SJZDP-II-I. (**A**) ^1^H-NMR spectra of SJZDP-II-I; (**B**) ^13^C-NMR spectra of SJZDP-II-I.

The polysaccharide fraction SJZDP-II-I was also characterized by 1D NMR spectroscopy and the chemical shift values compared with data from the literature [11,33,34,35,36]. Typically, the anomeric ^1^H signals of α-pyranoside are found over 5 ppm while those of β-pyranoside are at less than 5 ppm [37]. As shown in Figure 3, the ^1^H-NMR spectrum of polysaccharide fraction SJZDP-II-I contained five main anomeric protons at 5.11, 5.04, 5.02, 4.95 and 4.51 ppm, indicating the existence of both α- and β-configurations in the polysaccharide fraction SJZDP-II-I. The weak proton signal at 1.18 and 1.26 ppm indicated the presence of a methyl group of Rha in the fraction [35]. The ^13^C signals at 175.74 ppm corresponded to carbonyl carbon of unesterified α-1, 4-Gal*p*A, and the 103.14 ppm corresponded to C-1 of α-1, 4-Gal*p*A. Other signals such as 107.29 ppm corresponded to α- L-Ara*f*, while 106.80 ppm, 106.33 ppm and 104.29 ppm were corresponded to β-d-Gal*p* [33]. The signals at 16.75 ppm represented the C-6 of α-l-Rha [36]. These results suggested that fraction SJZDP-II-I was a typical pectic polysaccharide, with HG regions, RG-I regions and AG-I/AG-II side chains, which was consistent with the monosaccharide composition results.

### 2.5. SJZDP-II-I Treatment Didn’t Affect the Proliferation of SW480 Cells

**Figure 4:**
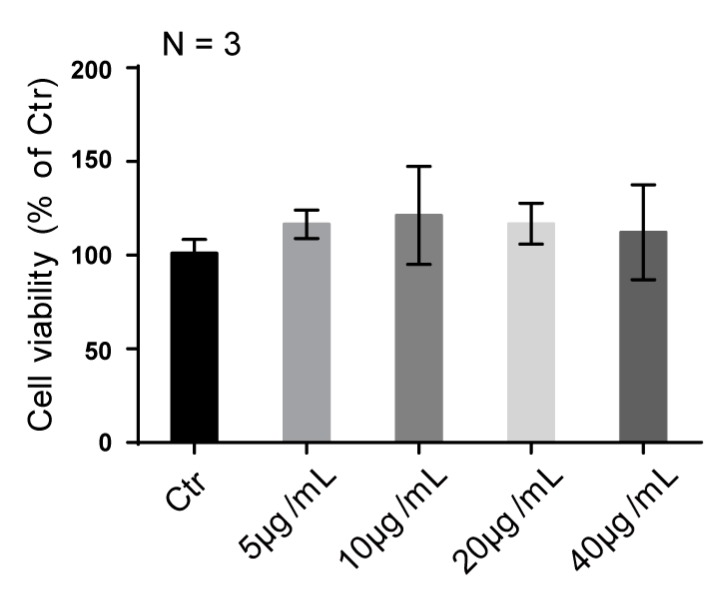
SJZDP-II-I treatment didn’t affect the proliferation of SW480 cells. Quantification shows no enhancement of cell proliferation and cytotoxicity of SJZDP-II-I in SW480 cells, with a concentration up to 40 µg/mL. Error bars indicate SD. Data are shown as mean ± SD of triplicates of one experiment, and the experiment was repeated 3 times.

Total SJZDP was reported to enhance cell proliferation [15], but no such activity of was found in the fraction SJZDP-II-I of this study. SW480 cells, derived from colon adenocarcinoma, exhibited similar proliferation rates after SJZDP-II-I treatment under different doses. Additionally, SJZDP-II-I exhibited no cellular toxicity at the concentration up to 40 µg/mL (Figure 4).

### 2.6. SJZDP-II-I Promoted the Antioxidant Defense of SW480 Cells

**Figure 5:**
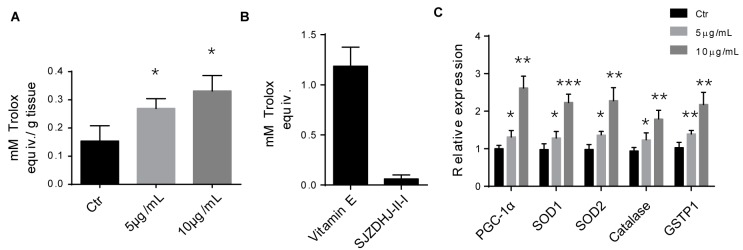
SJZDP-II-I treatment promoted the expression of antioxidant defense genes. (**A**) Quantification showed an increase of total antioxidant capability in SW480 cells after SJZDP-II-I treatment. Error bars indicated SD. * *p* < 0.05. *N* = 3; (**B**) In vitro assay displayed little antioxidant capacity of SJZDP-II-I when it was compared with Vitamin E. *N* = 3; (**C**) qRT-PCR showed robust increased mRNA levels of PGC-1α and antioxidant enzymes after SJZDP-II-I treatment in SW480 cells. Error bars indicated SD. * *p* < 0.05, ** *p* < 0.01, *** *p* < 0.001. *N* = 3.

SAs SJZD is widely used in gastrointestinal disorders, so it was reasonable to expect that SJZDP-II-I would affect the functions of these organs [1,2]. Total SJZD polysaccharides have been reported to promote the restoration of intestinal function by regulating intestinal homeostasis [2]. A growing body of evidence indicates that cellular oxidative stress and redox status are widely implicated in intestinal homeostasis, affecting cellular proliferation, apoptosis, metabolic processes, as well as the balance of intestinal microecology [21,38,39]. In this study, we found that SJZDP-II-I treatment could remarkably increase the total antioxidant capacity (TAC) of SW480 cells, but little antioxidant capacity of SJZDP-II-I itself was detected according to an in vitro assay (Figure 5). Thus, the enhanced TAC may result from the increased levels of antioxidants induced by the enhancement of gene expression in SW480 cells themselves, but not SJZDP-II-I. In order to clarify this, the expressions of representative antioxidant enzymes were evaluated, showing a robust increase of these enzymes after SJZDP-II-I treatment, as well as the master regulator of antioxidant defense—PGC-1α (Figure 5). These results indicated that SJZDP-II-I could protect SW480 cells from oxidative stress through the promotion of antioxidant enzymes’ expression, and suggested that SJZDP-II-I may participate in the regulation of intestinal homeostasis through the redox status control of the intestine.

## 3. Materials and Methods

### 3.1. Preparation of Polysaccharides

Sijunzi Decoction (SJZD) was derived from four herbs, *C. pilosula*, *G. uralensis*, *P. cocos* and *A. macrocephala*, in a ratio of 3:3:3:2 by weight. All prepared slices of these four herbs were purchased from Fushoutang Pharmacy (Chengdu, China). SJZD was used for the preparation of polysaccharides (SJZDP) as described previously [16]. Dried herbs (55 g) were soaked in distilled water (440 mL) for 30 min at room temperature and then extracted twice at 100 °C for 4 h. The extract fractions were combined and concentrated by rotary evaporation. The concentrated extracts were subjected for dialysis (3500 Da cut-off, Beijing Rui Da Heng Hui Science Technology Development Co. Ltd., Beijing, China) in running water for 2 days. After dialysis, the extracts were lyophilized, and named SJZDP.

The SJZDP was purified by anion exchange chromatography and gel filtration. First, the SJZDP (200 mg) was dissolved in distilled water (10 mL), filtered through a 0.45 µm filter and applied to a DEAE Sepharose (Fast Flow, FF) column (5 cm × 40 cm, Beijing Rui Da Heng Hui Science Technology Development Co. Ltd.). The neutral fraction was eluted with distilled water at 2 mL/min, while the acidic fractions were eluted with a linear NaCl gradient in water (0–1.5 M) at 2 mL/min. The elution profiles were monitored using the phenol-sulfuric acid assay [40]. The related fractions were pooled, dialyzed at cut-off 3500 Da against distilled water to remove of NaCl, and lyophilized. Second, the acidic fractions (50 mg) were dissolved in elution buffer (10 mM NaCl), filtered through a Millipore filter (0.45 μm), and subjected to gel filtration after application on a Sepharose 6FF column (2.5 cm × 100 cm, Beijing Rui Da Heng Hui Science Technology Development Co. Ltd., Beijing, China, and eluted with 10 mM NaCl at 1.0 mL/min. Fractions were pooled based on the elution profile, as determined by the phenol-sulfuric acid assay, dialyzed and lyophilized.

### 3.2. Chemical Compositions and Linkage Determination

The monosaccharide compositions of the fractions was determined by gas chromatography of the trimethylsilylated (TMS) derivatives of the methyl glycosides obtained after methanolysis with 3 M hydrochloric acid in anhydrous methanol for 24 h at 80 °C [41]. Mannitol was used as an internal standard. The TMS derivatives were analyzed by capillary gas chromatography on a Focus GC (Thermo Scientific, Milan, Italy). Glycosidic linkage elucidation was performed by methylation studies. Prior to methylation, the uronic acids at polymeric level were reduced with NaBD_4_ to their corresponding neutral sugars [42]. After reduction of the polymers, methylation, hydrolysis, reduction and acetylation were carried out [43]. The derivatives were analyzed by GC-MS using a GCMS-QP2010 (Shimadzu, Kyoto, Japan) attached to a Restek Rxi-5MS column (30 m; 0.25 mm i.d.; 0.25 µm film). The injector temperature was 280 °C, the ion source temperature 200 °C and the interface temperature 300 °C. The column temperature was 80 °C when sample was injected, then increased 10 °C/min to 140 °C, followed by 4 °C/min to 210 °C and then 20 °C/min to 300 °C. Helium was the carrier gas (pressure control: 80 kPa). The compound corresponding to each peak was characterized by the interpretation of the retention times and the characteristic mass spectra. The estimation of the relative amounts of each linkage type was related to the total amount of each monosaccharide type as determined by methanolysis [44]. 

### 3.3. Molecular Weight Determination

The homogeneity and molecular weight of the native purified polysaccharide fractions were determined by size exclusion chromatography on a Hiload^TM^ 16/60 Superdex^TM^ 200 prep grade column (GE Healthcare, Uppsala, Sweden) combined with the Äkta system (FPLC, Pharmacia Äkta, Amersham Pharmacia Biotech, Uppsala, Sweden). Dextran polymers (Pharmacia) of 10, 40, 70, 500 and 2000 kDa were used as calibration standards [45]. 

### 3.4. FT-IR and NMR Spectroscopy

Approximately 1 mg of polysaccharide sample was mixed with 150 mg of dried KBr powder, and pressed into a 1 mm thick disk for the analysis using a PerkinElmer FT-IR spectrophotometer (PerkinElmer, Waltham, MA, USA). The IR spectra were recorded in the range of 4000–400 cm^−1^ [46]. ^1^H-NMR and ^13^C-NMR spectra of two main polysaccharides fractions were recorded in D_2_O solution at 25 °C on an AV600 spectrometer (600 MHz, Bruker, Rheinstetten, Germany) after deuterium exchange three times by freeze-drying in D_2_O. 

### 3.5. Cell Culture

SW480 cells were routinely cultured in DMEM (Gibco, Waltham, MA, USA), supplemented with 10% FBS in an incubator under an atmosphere of 5% CO_2_ at 37 °C. Cells were plated in 6-well cell plates (1 × 10^6^ cells per well); 12 h after the polysaccharide supplement, cells were collected for gene expression detection and ABTS Radical Scavenging Activity Assay. Meanwhile, cell viability was assayed 12 h after the polysaccharide treatment in 96-well cell plate (5 × 10^4^ cells per well), using CCK-8 kit.

### 3.6. Cell Viability Assay

Cell viability was measured by Cell Counting Kit-8 (CCK-8) system (Dojindo, CK04-11, Minato-ku, Tokyo, Japan) according to the manufacturer’s instructions. Briefly, CCK-8 solution (10 µL per 100 µL of medium in each well) was added, and the plates were then incubated at 37 °C for 1 h. The absorbance of each well was read at 450 nm using a microplate reader (Thermo, Waltham, MA, USA).

### 3.7. Quantitative Realtime PCR

RNA extraction from the intestine and real time PCR for antioxidant genes detection were performed as previously reported [47]. Briefly, SW480 cells were lysed with Trizol Regent (Invitrogen, Waltham, MA, USA), and the total RNA was extracted from the tissues according to the manufacturer’s instructions. The quality of RNA was assessed by agarose gel and the concentration was measured with a spectrophotometer (NanoDrop 2000, Thermo Scientific, Shanghai, China). Total RNA was subjected to reverse transcription with a reverse transcriptase, according to the manufacturer’s instructions (Fermentas, Waltham, MA, USA). Quantitative real-time PCR was performed using the Bio-Rad CFX96 system, and the relative gene expression was normalized to the internal control actin. Primer sequences for SYBR Green probes of the target genes are described in Table 3.

### 3.8. ABTS Radical Scavenging Activity Assay

The total antioxidant capacity was evaluated using the ABTS method (Beyotime, Shanghai, China). ABTS radical cation (ABTS^+^) solution was produced by reacting ABTS stock solution with 2.45 mmol/L potassium persulfate in the dark at room temperature for 12–16 h before use, then it was diluted with 80% ethanol to adjust the absorbance to 0.70 ± 0.05 at 734 nm. After that, 10 µL of sample or Trolox standard was added into 200 µL of diluted ABTS^+^ solution. The absorbance at 734 nm was measured after 2–6 min in the dark at room temperature. Trolox, a water-soluble analogue of vitamin E, was used as the reference standard to prepare a calibration curve with a concentration range of 0.15–1.5 mM. Results were expressed as mmol/g Trolox equivalent antioxidant capacity.

### 3.9. Statistical Analysis

Data represent the means and SD. One-way ANOVA and post hoc tests were performed for all statistical significance analysis using GraphPad Prism software (GraphPad Software, Inc., La Jolla, CA, USA). * *p* < 0.05, ** *p* < 0.01, *** *p* < 0.001.

## 4. Conclusions

In summary, a purified polysaccharide from SJZD was obtained by using ion exchange chromatography and gel filtration. Structural analysis by FT-IR and NMR identified SJZDP-II-I as a typical pectic polysaccharide. Moreover, this SJZDP-II-I was shown to effectively promote the antioxidant defense capability in SW480 cells, which will be valuable for further research and applications.

## Figures and Tables

**Table 1:** Monosaccharide compositions (mol %) and Mw of polysaccharide fractions SJZDP-I-I, SJZDP-I-II and SJZDP-II-I, isolated from SJZDP.

	SJZDP-I-I	SJZDP-I-II	SJZDP-II-I
Ara ^a^	51.2	18.9	32.8
Rha ^a^	2.3	1.7	5.0
Fuc ^a^	2.1	n.d.	0.4
Xyl ^a^	n.d.	2.4	0.4
Man ^a^	3.7	1.4	0.4
Gal ^a^	23.6	4.7	40.5
Glc ^a^	11.2	66.9	1.5
GlcA ^a^	0.8	n.d.	1.5
GalA ^a^	5.2	4.0	17.4
Mw (kDa)	351.0	16.9	171.9

n.d. not detected. ^a^ mol % related to total content of the monosaccharides arabinose (Ara), rhamnose (Rha), fucose (Fuc), mannose (Man), galactose (Gal), glucose (Glc), Glucuronic acid (GlcA) and Galacturonic acid (GalA).

**Table 2:** The glycosidic linkage (mol %) present in the fraction SJZDP-II-I isolated from SJZDP.

	Linkage Type	mol %
Ara	T*f*	22.7
	1→5*f*	3.7
	1→3,5*f*	6.4
Rha	T*p*	0.4
	1→2*p*	1.8
	1→2, 4*p*	2.8
Gal	T*p*	2.4
	1→4*p*	10.7
	1→3*p*	2.2
	1→6*p*	5.1
	1→3, 6*p*	20.1
Glc	T*p*	1.5
GlcA	T*p*	1.5
GalA	T*p*	0.9
	1→4*p*	16.5

**Table 3:** qRT-PCR primers for antioxidant defense genes.

SOD1	Fr 5′-CAAGCGGTGAACCAGTTGTG-3′
	Rv 5′-TGAGGTCCTGCACTGGTAC-3′
SOD2	Fr 5′-GCCTGCACTGAAGTTCAATG-3′
	Rv 5′-ATCTGTAAGCGACCTTGCTC-3′
Catalase	Fr 5′-ACCCTCTTATACCAGTTGGC-3′
	Rv 5′-GCATGCACATGGGGCCATCA-3′
GSTP1	Fr 5′-ATGCCACCATACACCATTGTC-3′
	Rv 5′-GGGAGCTGCCCATACAGAC-3′
PGC-1α	Fr 5′-CTCCCTGTGGATGAAGACGG-3′
	Rv 5′-GCAAATCACAATCACAGGAT-3′

SOD, Superoxide dismutase; GSTP, Glutathione S-transferase Pi; PGC-1a, Peroxisome proliferator-activated receptor gamma coactivator 1-alpha.
